# How Postural Muscle Senses Disuse? Early Signs and Signals

**DOI:** 10.3390/ijms21145037

**Published:** 2020-07-16

**Authors:** Boris S. Shenkman

**Affiliations:** Myology Laboratory, Institute of Biomedical Problems RAS, 123007 Moscow, Russia; bshenkman@mail.ru

**Keywords:** soleus muscle, disuse atrophy, AMPK, NO, mechanotransduction, ROS, E3-ubiquitin ligases, myosin phenotype

## Abstract

A mammalian soleus muscle along with other “axial” muscles ensures the stability of the body under the Earth’s gravity. In rat experiments with hindlimb suspension, zero-gravity parabolic flights as well as in human dry immersion studies, a dramatic decrease in the electromyographic (EMG) activity of the soleus muscle has been repeatedly shown. Most of the motor units of the soleus muscle convert from a state of activity to a state of rest which is longer than under natural conditions. And the state of rest gradually converts to the state of disuse. This review addresses a number of metabolic events that characterize the earliest stage of the cessation of the soleus muscle contractile activity. One to three days of mechanical unloading are accompanied by energy-dependent dephosphorylation of AMPK, accumulation of the reactive oxygen species, as well as accumulation of resting myoplasmic calcium. In this transition period, a rapid rearrangement of the various signaling pathways occurs, which, primarily, results in a decrease in the rate of protein synthesis (primarily via inhibition of ribosomal biogenesis and activation of endogenous inhibitors of mRNA translation, such as GSK3β) and an increase in proteolysis (via upregulation of muscle-specific E3-ubiquitin ligases).

## 1. Introduction. When the Support is withdrawn. EMG Drop

A mammalian soleus is a very active muscle. It is active for at least 11 h a day [1]. Along with other “axial” muscles (for example, back muscles), it provides stability for the mammalian body in the gravitational field of the Earth. The contractile activity of a postural muscle is determined by the two main biomechanical factors that affect the motor system of animals and humans on our planet: (1) axial loading and (2) ground reaction force. Both factors exert their influence under conditions of Earth’s gravity and are eliminated under conditions of weightlessness. Axial loading is significantly reduced to almost the same extent in both dry immersion and bedrest models of simulated microgravity. At the same time, ground reaction force during bedrest is redistributed over the surface of the body and reduced but not completely eliminated, while under conditions of dry immersion when the human body is “suspended” in water the ground reaction force is virtually absent [2,3,4]. In a well-known model of rat hindlimb suspension (HS), the effect of both axial loading and ground reaction force on the rear part of the animal body is negligible. Interestingly, the physiological effects of these factors on the mammalian soleus muscle are different. Under conditions of human dry immersion, an electrical (and, of course, mechanical) activity of the soleus muscle can be significantly increased if mechanical pressure (mechanical stimulation) is applied to the foot [5,6]. This demonstrates that even when the axial loading is dramatically diminished, the influence of support on the foot can maintain a contractile activity of this muscle. Axial loading, i.e., sustaining the body weight, obviously exerts a resistive effect on the contraction of soleus muscle fibers.

Of particular interest are the physiological consequences of eliminating both the axial mechanical loading and the ground reaction force. In rat experiments with HS and short-term zero-gravity parabolic flights as well as in human dry immersion studies, a dramatic decrease in the electromyographic (EMG) activity of the soleus muscle has been repeatedly shown [7,8,9]. Thus, the elimination of gravity-dependent mechanical influences leads to the “switching off” of the electrical and, accordingly, mechanical activity of the soleus muscle, the main anti-gravity muscle of mammals.

Long-term consequences of chronic inactivation of the soleus muscle during gravitational unloading include a deep loss of muscle stiffness (atonia) [2,10], a change in myosin phenotype (slow-to-fast fiber type transition) (for review see [11]) and a dramatic decrease in muscle weight/protein mass (atrophy) [12].

One-day mechanical unloading results in a significant increase in mRNA expression of muscle-specific E3-ubiquitin ligases MuRF-1 and MAFbx/atrogin-1 as well as MuRF-1 protein content in rat soleus muscle [13]. At the same time, a decrease in protein synthesis by more than 40% and more than a twofold decrease in the content of 18S and 28S ribosomal RNAs are demonstrated in rat soleus following 24-h unloading [14]. These events are accompanied by a significant reduction in the expression of myh7 gene encoding slow isoform of myosin heavy chains (MyHC I (β)) [15,16].

In this regard, it is important to understand how intracellular signaling pathways receive information about the reduction/termination of contractile activity and, based on this information, rearrange their work, i.e., what metabolic, ionic and mechanobiological events (directly caused by a drastic reduction in the soleus contractile activity) trigger a shift in the intracellular balance of anabolic and catabolic signaling pathways towards the predominance of destructive processes.

A natural consequence of the termination of contractile activity of muscle fibers is a change in the balance of purine nucleotides (ATP/ADP/AMP) towards the accumulation of ATP [17] and a 25–35% increase in the glycogen content after 24 h of unloading [18]. Some authors consider the accumulation of reactive oxygen species (ROS) as one of the key factors in unloading-induced muscle remodeling [19]. At the same time, nitric oxide (NO) levels (for review see [20]), as well as the content of neuronal nitric oxide synthase (nNOS) [21] in the sarcolemma of the soleus muscle fibers, are reduced during unloading. It can be assumed that when the nature of the contractile activity of the postural muscle changes, high-energy phosphates and glycogen can act as metabolic signals that affect the state of regulatory networks that determine changes in protein metabolism and myosin phenotype. The same role may be performed by ROS, including NO.

One of the early and, possibly, triggering events of mechanical unloading is the change in the ion balance as a result of the cessation of the wave of depolarization. In this case, disturbances in the function of Na^+^, K^+^ - ATPase, sodium, and chloride channels occur followed by changes in the nature of calcium flux. In 1995 it was predicted [22] and in 1999–2001 it was shown that 48-h HS leads to accumulation of calcium ions in the myoplasm of soleus muscle fibers [23,24]. A possible signaling role of this phenomenon was marked by S. Kandarian [25]. However, only in recent years has it been found that an excess of calcium ions not only contributes to the activation of proteolytic enzymes (for review see [26]) and the regulation of expression of a number of key genes, including isoforms of MyHC [27], but also participates in the regulation of mitochondrial function and metabolism of free radicals [28]. Thus, calcium ions can also be classified as a metabolic trigger for gravitational unloading.

Thus, adenine nucleotides (ATP, ADP), glycogen, reactive oxygen species, nitric oxide, and calcium ions are all hypothetical signaling messengers that play an important role during soleus muscle unloading. It is also important to note that the sarcolemma of muscle fibers is rich in mechanosensory molecules, the most studied of which are integrins and mechanosensitive/stretch-activated ion channels (SAC). With the cessation of the contractile activity of the soleus muscle, we should expect a corresponding cessation of mechanical stimuli sensed by the mechanosensory molecules. This yet unexplored impairment in the function of mechanosensors can also serve as a trigger changing the balance in the activity of anabolic and catabolic signaling pathways.

In this paper, several metabolic events at the early stage of the cessation of soleus muscle contractile activity due to unloading (mainly up to 24 h) will be reviewed. These metabolic events will be linked in a way to present a logical and consistent picture that would allow readers not only to see the sequence of signaling reactions but also to identify the atrophic pattern of this chain of regulatory events.

## 2. Energy Supply Associated Events: AMP/ADP/ATP Ratio and Signaling Consequences. AMPK dephosphorylation

**Figure 1:**
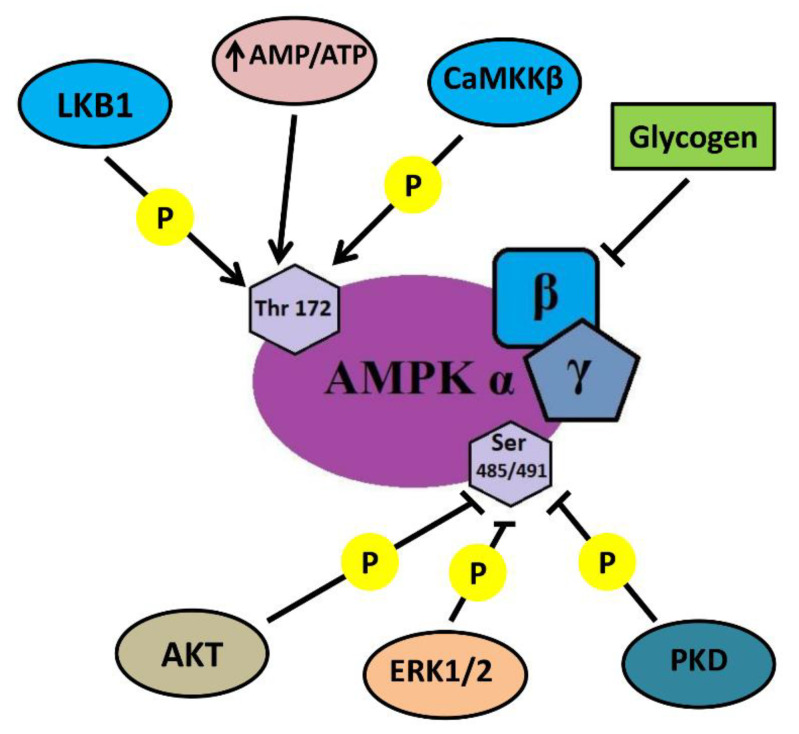
Upstream influences on AMP-activated protein kinase (AMPK). Liver kinase B1 (LKB) and calcium-calmodulin kinase II (CaMKKII) can phosphorylate AMPK at Thr172 site and increase the activity of its reaction center. The phosphorylation of this site is also facilitated by AMP. Phosphorylation at Ser 485/491 site reduces the level of Thr172 phosphorylation and, accordingly, AMPK activity. AMPK Ser 485/491 phosphorylation can be mediated by AKT (protein kinase B), mitogen-activated protein kinase (ERK1/2), and protein kinase D (PKD). An interaction with glycogen via the β subunit also reduces the level of AMPK (Thr172) phosphorylation and its activity. Thus, AMPK activity is the result of an interaction of both activating and inhibiting influences.

In contracting skeletal muscle myofibrillar apparatus consumes energy derived from adenosine triphosphate (ATP). To sustain muscle activity, ATP needs to be regenerated by energy systems (aerobic oxidation, anaerobic glycolysis) so that the ratio of ATP/AMP is maintained in a state of dynamic equilibrium. When muscle stops working, we should expect a natural shift in this ratio with the accumulation of ATP in the myoplasm. Some signs of such accumulation in rat soleus muscle after HS were observed by Wakatsuki et al. [17] using NMR spectroscopy. It should also be expected that a direct consequence of ATP accumulation and a corresponding decrease in AMP can be a decrease in the phosphorylation level of AMP-activated protein kinase (AMPK). Until recently, data on the effect of gravitational unloading on the level of AMPK Thr172 phosphorylation remained contradictory. After 14 days of HS, some authors reported an increase in AMPK Thr172 phosphorylation in rat soleus muscle [29], while others demonstrated a decrease in the phosphorylation level of this kinase [30]. No significant changes in AMPK Thr172 phosphorylation were found in mouse soleus after 10-day unloading [31]. However, until 2015, there were no reports on the effect of short-term simulated microgravity on the level of AMPK phosphorylation. Only in 2015 Vilchinskaya and co-authors from our laboratory showed a significant decrease in the phosphorylated forms of AMPK in human soleus muscle after 3-day dry immersion [32]. A decrease in this indicator was also found in rat soleus muscle after 24 and 12 h of HS [16,33,34]. Thus, AMPK dephosphorylation in the soleus muscle is a well-established event within the first day of unloading. At this point, an obvious question arises about the causes and signaling consequences of this phenomenon. The initial hypothesis about the role of changes in the balance of adenine nucleotides in the process of AMPK dephosphorylation required verification. It was previously shown that β-guanidinopropionic acid (β-GPA) (a competitive creatine phosphokinase inhibitor) administration can lead to a greater expenditure of ATP and an increase in AMPK phosphorylation [35]. As an increase in AMPK phosphorylation in rat soleus muscle requires a relatively long period of supplementation with AMPK stimulators [36], a modified protocol that combined daily administration of β-GPA for 6 days before HS and 1 day during HS was applied [37]. β-GPA administration resulted in complete prevention of 1-day-unloading-induced AMPK Thr172 dephosphorylation in rat soleus muscle [38]. So, we can assume that at least one of the factors leading to AMPK dephosphorylation is a change in the balance of adenine nucleotides (Figure 1). Another factor that can affect the level of AMPK Thr172 phosphorylation is a significant accumulation of glycogen in rat soleus during the first three days of HS [18]. It is known that there is a site on the β-subunit of AMPK that has an affinity for glycogen which can allosterically inhibit phosphorylation of the activating site on the α-subunit of AMPK [39,40]. It is possible that this mechanism could enhance the level of AMPK dephosphorylation during the first day of unloading (Figure 1). To date, this assumption has not received any experimental confirmation. At the same time, there is a Ser485/491 site on the α-subunit of the AMPK molecule, phosphorylation of which can lead to dephosphorylation of the Thr172 site and subsequent decrease in the enzymatic activity of AMPK [41].

We showed, for the first time, a sharp increase in AMPK Ser485/491 phosphorylation following 24-h HS [16]. This increase in the inhibitory AMPK phosphorylation was accompanied by an increase in phosphorylation of protein kinase D (PKD) [16], which is considered to be responsible for the phosphorylation of AMPK at Ser485/491 [42]. To find out whether PKD activation impacts AMPK phosphorylation under 1-day HS, an additional experiment was performed using a specific PKD inhibitor CRT 0066101. The use of the PKD inhibitor completely prevented the unloading-induced decrease in AMPK Thr172 phosphorylation (unpublished observation). Thus, in these experiments, Vilchinskaya et al. [16] found two mechanisms that contribute to AMPK dephosphorylation at Thr172: the change in the balance of adenine nucleotides and the activation of protein kinase D (Figure 1). The nature of the first mechanism is generally clear: an inactive muscle does not consume ATP. As for the PKD activity, much about this mechanism remains unclear. It is known that an increase in PKD phosphorylation is observed when AMPK activity is reduced or absent due to knockout [43]. It can be assumed that the dephosphorylation of AMPK due to the changes in the balance of adenine nucleotides during unloading could contribute to the activation of PKD, which, in turn, could further increase the degree of AMPK dephosphorylation. This hypothesis has yet to be tested.

## 3. Mystery of p70S6K Hyperphosphorylation. Exercise-Rest Signaling Transition. Soleus Unloading as the “prolonged rest”. Anabolic-Induced Catabolic Attack

What are the signaling consequences of AMPK (Thr172) dephosphorylation (and inactivation) in the rat soleus muscle at the early stage of unloading? Recently, a paradoxical increase in p70S6K (Thr389) phosphorylation (p70S6K is one of the mTORC1 substrates) in rat soleus has been demonstrated within 24 h of HS [33,34]. These data are consistent with a report by You et al., 2015 in which similar changes in mouse soleus were shown following 3-day limb immobilization [44]. You et al., 2015 do not explain possible mechanisms of such a paradoxical increase in mTORC1 activity while the rate of protein synthesis is reduced and suggest that mTORC1 activation, in this case, is an attempt of the physiological system to compensate for a disuse-induced decrease in the muscle protein content [44]. An increase in the level of p70S6K phosphorylation was also observed during the first days after denervation in mouse fast hindlimb muscles [45,46]. Quy and co-authors suggest that the activation of mTORC1/p70S6K pathway and subsequent suppression of autophagy during the initial period of denervation is associated with the activation of proteolysis and subsequent accumulation of amino acids, which are known to stimulate mTORC1 [45]. Tang et al., 2014 also link mTORC1 activation with the signaling mechanisms of the ubiquitin-proteasome system [46]. The use of a selective mTORC1 inhibitor rapamycin led to a significant reduction in the level of muscle atrophy after 14-day denervation. However, these hypotheses do not allow us to explain the increase in p70S6K phosphorylation after 24 h of unloading. It is very unlikely that within such a short period of time, enough amino acids for mTORC1 activation are accumulated.

**Figure 2:**
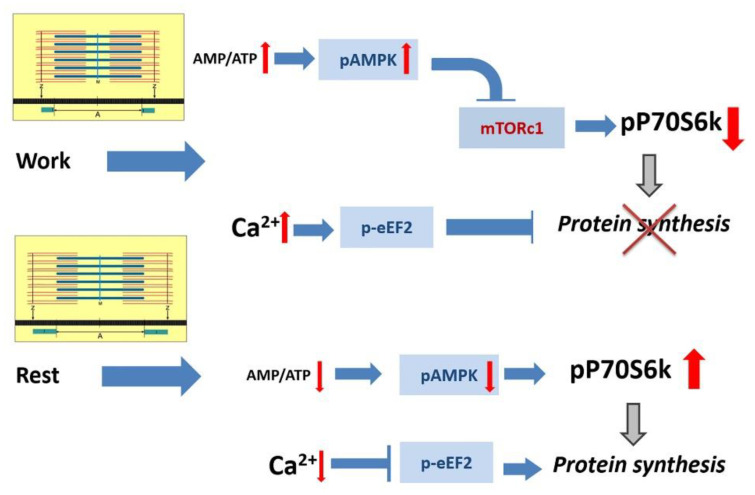
Suppression of the anabolic processes during muscle contractile activity and an upregulation of the anabolic processes after exercise. A contraction-induced increase in the concentration of AMP and calcium ions leads to the activation of AMP-activated protein kinase (AMPK) and eukaryotic elongation factor 2 (eEF2), respectively. AMPK suppresses the activity of mTORC1, and phosphorylated eEF2 (due to an increased calcium concentration) contributes to the inhibition of polypeptide chain elongation. After muscle contraction, AMPK activity decreases, and under reduced myoplasmic calcium concentration, dephosphorylated eEF2 stimulates polypeptide chain elongation enhancing the rate of muscle protein synthesis.

One explanation for the transient increase in p70S6K phosphorylation during the initial period of unloading may be associated with one important pattern of the activation of anabolic processes in a skeletal muscle, the significance of which was highlighted by M. Rennie: “muscle stops building when it’s working” [47]. Indeed, during muscle contraction, anabolic signaling pathways and protein synthesis are never activated (Figure 2). First of all, this applies to the mTORC1/p70S6K pathway. There are mechanisms for suppressing the mTORC1/p70S6K signaling pathway and mRNA translation during muscle activity. These mechanisms are associated with eukaryotic elongation factor 2 (eEF2) and AMPK [48,49]. During muscle activity, these signaling molecules are activated by increased concentrations of calcium ions and AMP, respectively, leading to the inhibition of polypeptide chain elongation and mTORC1 activity [50]. After the cessation of muscle contractions, the activity of these enzymes naturally decreases, and the anabolic pathways become activated [51,52]. We assume that similar processes occur when rat soleus muscle is inactivated due to unloading.

It is known that AMPK activity in soleus muscle is quite high [41], however, muscle unloading leads to a rapid dephosphorylation/inactivation of this enzyme (see above). As the inhibitory effect of AMPK on mTORC1-signaling is eliminated, one should observe an increase in the level of p70S6K phosphorylation. This hypothesis was tested in an experiment with the activation of AMPK during the first day of unloading with a well-known specific AMPK activator AICAR [13]. In this study, a 1-day unloading-induced increase in p70S6K phosphorylation in the rat soleus muscle was not observed when rats were treated with AICAR. Thus, highly likely, we can say that the dephosphorylation of AMPK at the initial stage of unloading is one of the important conditions for the increased level of p70S6K phosphorylation. Another possible cause for the increase in p70S6K phosphorylation during early unloading may be an increase in ceramide concentration. Recently, Bryndina’s group found that 12 h unloading can lead to ceramide accumulation in the rat soleus muscle [53,54]. At the same time, in vitro experiments showed that an addition of ceramide can significantly increase the level of p70S6K phosphorylation as well as insulin receptor substrate (IRS-1) Ser636/639 phosphorylation [55]. It is assumed that the negative IRS-1 Ser636/639 phosphorylation is caused by p70S6K hyperphosphorylation [56] due to the effect of ceramide [55]. The reasons for ceramide accumulation during the first hours of unloading [53] remain unclear. However, it is noteworthy that the use of AICAR (AMPK activator) could successfully inhibit ceramide accumulation [57].

An increased level of glycogen that was earlier detected in rat soleus muscle during the first days of unloading [18] can also directly stimulate p70S6K phosphorylation without AMPK participation [58].

**Figure 3:**
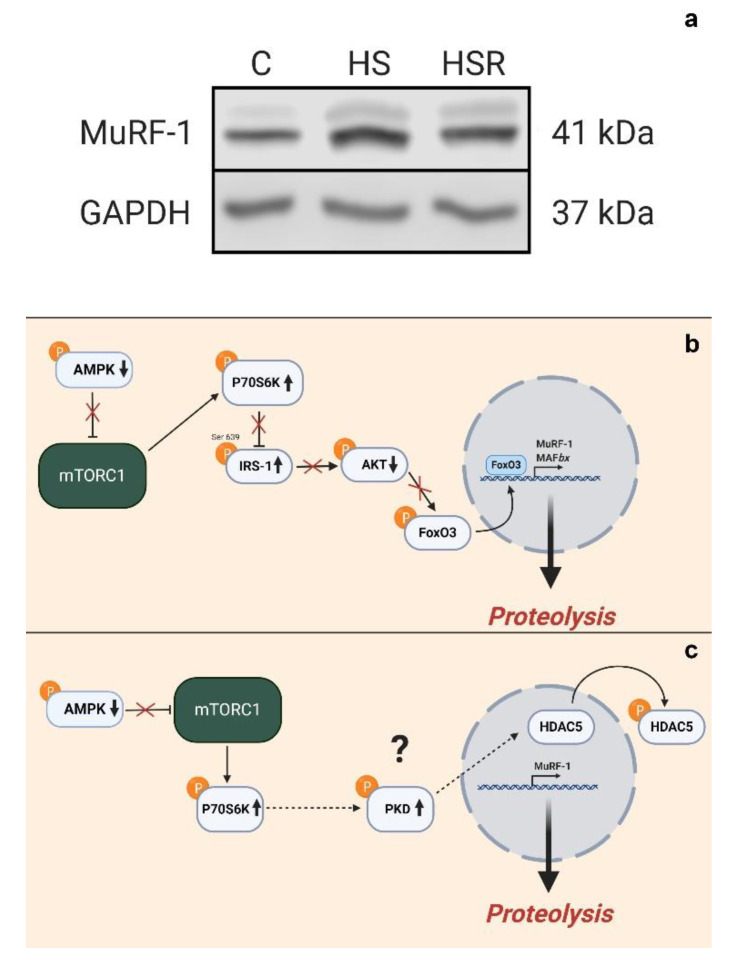
Causes and signaling consequences of the increased phosphorylation of ribosomal protein S6 kinase (p70S6K) during the first day of unloading (according to Belova et al. [13]). (**a**) Experimental confirmation of the role of increased p70S6K (Thr389) phosphorylation in the early increase in the expression of E3-ubiquitin ligases. During the first day of unloading, there is an increase in protein expression levels of MuRF-1. When rapamycin (mTORC1 inhibitor) reduces the level of p70S6K phosphorylation, the increase in MuRF-1 expression is not detected. (**b**) An initial hypothesis suggesting the role of increased p70S6K phosphorylation in the regulation of MuRF-1 expression via IRS-1 downregulation (Hsieh et al. [55]). AMPK dephosphorylation leads to the mTORC1 unblocking and increased p70S6K phosphorylation, which, in turn, phosphorylates IRS-1 at Ser639 thereby inhibiting downstream components of IGF-1- signaling. These events can lead to FOXO3 nuclear and increased MuRF-1expression. However, it turned out that the blocking of this signaling cascade does not depend on p70S6K phosphorylation. (**c**) A working hypothesis suggesting the role of increased p70S6K phosphorylation in stimulating MuRF-1 expression through the removal of the histone deacetylase 5 (HDAC5)-related epigenomic blockade. As shown in the experiment with rapamycin treatment during 24-h hindlimb unloading, the increased p70S6K phosphorylation leads to the HDAC5 nuclear export and resulting in the release of murf1 gene and increased expression of the E3-ubiquitin ligase MuRF-1. Protein kinase D may be involved in this process (Vilchinskaya et al. [16]).

Interestingly, rapid development of the atrophic process in mouse soleus during three-day hindlimb immobilization can be prevented by rapamycin [44] and sestrin overexpression [59], which leads to a decrease in the level of p70S6K phosphorylation. This anti-atrophic effect of mTORC1 inhibition appears to be associated with the prevention of autophagy suppression [59]. In a study from our laboratory by Belova and co-authors (2019), a different mechanism of the atrogenic effect of increased p70S6K phosphorylation was identified [13]. After 24 h of HS, hyperphosphorylation of p70S6K (Thr389) was accompanied by a sharp increase in the mRNA expression and protein abundance of E3-ubiquitin ligase MuRF1 [13] (Figure 3a). To find out whether these phenomena are related, rats were treated with rapamycin (a specific mTORC1 inhibitor) during 1-day unloading which resulted in a decrease in p70S6K phosphorylation to the level of vivarium control. This effect led to changes in a number of other signaling markers. Rapamycin treatment completely prevented an increase in the expression of E3-ubiquitin ligases, primarily MuRF1. Based on the results of Hsieh et al. [55], we suggested [13] that increased p70S6K phosphorylation could lead to increased expression of the E3-ubiquitin ligases via negative IRS1 S636-639 phosphorylation leading to impaired signal transduction to AKT, which, in turn, could result in FOXO3A dephosphorylation, translocation to the nucleus and activation of murf1 gene transcription (Figure 3b). However, it turned out that the total content of IRS-1 significantly decreased following 1-day HS independently of rapamycin treatment. The levels of AKT and FOXO3A phosphorylation were also rapamycin-independent. So, despite all necessary conditions for increased MuRF1 mRNA expression in the 1-day HS+rapamycin group were met, MuRF1 expression remained at the level of vivarium control. According to our working hypothesis dependence of MuRF1 expression on the level of p70S6K phosphorylation could be carried out via mechanisms of epigenetic regulation. It was found that rapamycin treatment prevented a 24-h HS-induced decrease in histone deacetylase-5 (HDAC5) nuclear content [13]. As MuRF1 expression can be blocked with HDAC5 nuclear import and activated with HDAC5 nuclear export [60], it can be assumed that some unknown p70S6K-dependent protein kinase can phosphorylate HDAC5 and promote its nuclear export thereby releasing MuRF1 expression. When the level of p70S6K phosphorylation is decreased due to rapamycin treatment, these events may not occur (Figure 3c). It is possible that, in this case, nuclear-cytoplasmic traffic of HDAC5 can be mediated by protein kinase D (PKD) [16]. Thus, the experiment with the administration of rapamycin during 24-h unloading demonstrated that due to p70S6K hyperphosphorylation, mRNA expression of E3-ubiquitin ligases in the rat soleus muscle is upregulated [13]. This upregulation is a consequence of epigenetic unblocking of genes encoding the key muscle-specific E3-ubiquitin ligases.

The results of the experiment help to depict a general picture of the state of signaling processes in rat soleus muscle during the first hours of unloading. Following the deactivation of the majority of motor units of the soleus muscle, the signaling processes enter a state resembling a “post-exercise rest and recovery” state. However, the duration of this “rest period” for the soleus muscle is unusually long. And such a large number of soleus muscle fibers in normal life are not inactivated. Therefore, it is not surprising that prolonged (for several hours) activation of the anabolic regulator leads to a corresponding activation of the expression of the key regulators of proteolysis. This response may be related to the maintenance of intracellular homeostasis. 

## 4. E3-Ubiquitin Ligase Expression Control, IRS-1 Degradation. ROS as the Metabolic Triggers. Resting Membrane Potential Shift and its Consequences

**Figure 4:**
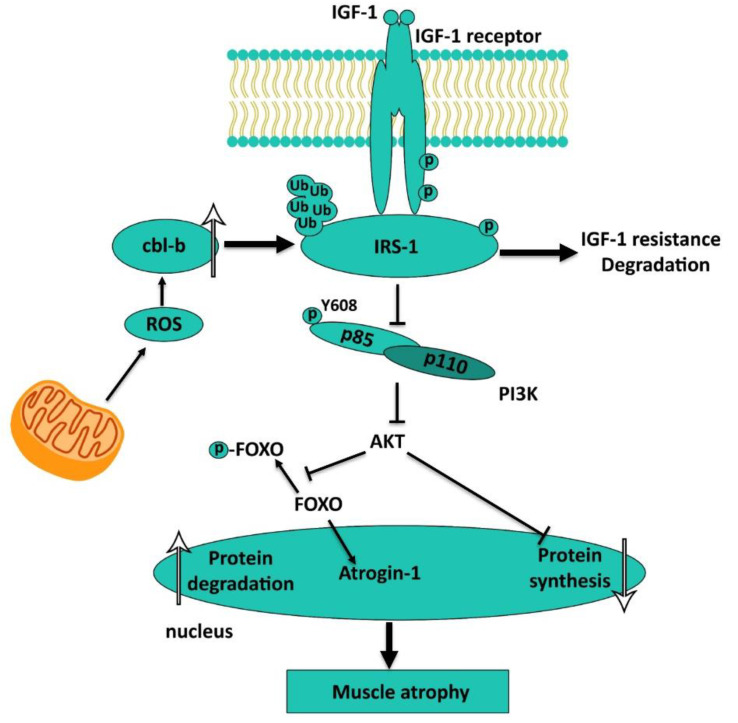
ROS-dependent mechanism of partial elimination of IRS-1 and IGF-1 resistance, contributing to the increased expression of E3-ubiquitin ligases [65,66]. During the first days of unloading (according to [16], during the first 24 h) under the action of mitochondria-derived ROS, the expression of E3-ubiquitin ligase cbl-b increases, leading to IRS-1 ubiquitylation and subsequent degradation. As a result, signal transduction from insulin/IGF-1 to AKT gets impaired, and AKT becomes underphosphorylated. This leads to the insufficient FOXO3 phosphorylation, which induces FOXO3 nuclear translocation increasing the expression of muscle-specific E3-ubiquitin ligases.

Effective transcription of E3-ubiquitin ligases’ genes is possible only when the corresponding transcription factors are imported into the nuclear space and activated. These are, primarily, factors of the FOXO family and myogenin. It is well kno wn that the nuclear import of the FOXO factors is mediated by their dephosphorylation under decreased AKT activity [61,62]. As previously shown, blocking the signal from insulin and/or IGF1 receptors to AKT during gravitational unloading is achieved by ubiquitinylation and subsequent degradation of IRS1. IRS1 degradation was detected in rat skeletal muscles after both chronic hindlimb suspension (14, 28, 38, 56 days) [29,30,63,64] and short-term periods of unloading (5 days), as well as after 16-day space flight [65]. Nakao et al., 2009 suggested that IRS1 degradation during unloading is mediated by ubiquitinylation, which is associated with E3-ubiquitin ligase cbl-b. Recently, this group of authors, led by professor T. Nikawa, have reported strong evidence in favor of their hypothesis linking cbl-b expression to the accumulation of reactive oxygen species (ROS) in muscle fibers under unloading conditions [66]. These authors suggested that the accumulation of ROS under unloading conditions leads to the increased cbl-b expression and subsequent ubiquitinylation and degradation of IRS1. This IRS1 degradation can contribute to impaired IGF-1/insulin-dependent signaling and reduced AKT phosphorylation at Thr308 and Thr473 activating sites. This, in turn, leads to the insufficient phosphorylation of FOXO3 protein and its translocation into the nucleus, followed by the activation of gene promoters of the key E3-ubiquitin ligases (Figure 4). All these events are confined within 5-7 days of unloading [65]. In our paper (Belova et al., 2019), it was first established (see above) that a significant IRS-1 degradation in rat soleus muscle is observed after 24 h of unloading [13]. Unfortunately, data on the cbl-b expression in the soleus muscle fibers during the first day of unloading are not available in the literature. However, a group led by L. Gorza demonstrated an increase in ROS, indirectly measured by tropomyosin disulfide bonds, in rat soleus as early as after 6-h HS [21]. Thus, at the initial stage of muscle unloading ROS appear to stimulate cbl-b expression, IRS-1 degradation, AKT dephosphorylation, and FOXO3 nuclear translocation according to the hypothesis of T. Nikawa. In this case, the expression of E3-ubiquitin ligases (MuRF-1 and MAFBx/atrogin-1) gets enhanced, whilst HDAC5 excluding from the myonuclei.

During prolonged hypokinesia, the accumulation of ROS can significantly contribute to the development of muscle atrophy [19,67]. However, with short-term disuse, the contribution of this factor to the development of soleus muscle atrophy seems not so great, as evidenced by the low anti-atrophic effect of antioxidants [68]. However, experiments on the impact of ROS on the IRS-1 degradation provide grounds for the hypothesis about the role of this mechanism in the upregulation E3-ubiquitin ligases during the first day unloading. Moreover, under unloading conditions, ROS can trigger the expression of E3-ubiquitin ligases via the NFκB signaling pathway [69]. Summing up the data, ROS can be attributed to the triggers for the signaling pathways remodeling in skeletal muscle fibers during gravitational unloading. In this regard, one of the most interesting questions is the question about the mechanism of ROS production at the early stage of gravitational unloading. There are two main sources of ROS in muscle fibers: 1) mitochondria that form ROS during incomplete coupling of oxidation and phosphorylation [70] and NADPH-oxidase-2 (NOX-2), which localizes in the sarcolemma and internal membranes (for refs see [71]). During long-time exposure to unloading, an increased formation of ROS in skeletal muscle is observed [72]. There is less data on the accumulation of ROS in the first hours/days of unloading and these data are contradictory. On the one hand, it has recently been shown that during the first days of unloading in mouse gastrocnemius and soleus muscles, the indicators of respiratory control showing the degree of respiration and phosphorylation coupling do not change [73]. It is clear that in this case, we should not expect an intensive accumulation of the free radicals of mitochondrial origin. On the other hand, in the study by Gorza’s group, an increased level of tropomyosin di/trimerization after 6 h of unloading, indicating a significant accumulation of ROS, was eliminated by the use of mito-TEMPO (mitochondria-targeted antioxidant) [21]. This result can be considered as an argument in favor of the intensification of the mitochondrial production of free radicals already at the initial stage of unloading. As for NOX-2, the literature discusses its activation via the mechanism of purinergic signaling triggered by sarcolemma depolarization through the sensors of the action potential, dihydropyridine receptors [74].

Back in the 1970s, it was discovered that after exposure to real microgravity, a subthreshold decrease in the resting membrane potential (RMP) is observed in rat soleus muscle [for review see 26]. A similar phenomenon is detected in rat soleus muscle during hindlimb unloading of various durations [75,76,77]. In particular, a decrease in RMP was registered after 6 and 12 h of HS [34,78]. Different authors have different views on the causes of the decline in RMP. Pierno and colleagues suggested that the main reason for the RMP decrease is a change in the activity of cClC-1 chloride channels [75]. However, it turned out that the blockade of the electrogenic function of the α2 subunit of Na^+^, K^+^-ATPase with a 1 µM of ouabain resulted in reduced RMP in muscle fibers of intact rats, but did not lead to a further decrease in RMP in the unloaded rats [79]. Thus, it is highly likely that it is the decrease in the electrogenic function of the α2 subunit of Na^+^/K^+^-ATPase that causes the subthreshold decrease in RMP already at the early stage of unloading. Recently, we have shown that the subthreshold depolarization in rat soleus muscle fiber membranes during 12-h HS is effectively prevented by the use of AMPK activator AICAR [80]. Therefore, one of the reasons for the sub-threshold depolarization of the membrane at the initial stage of unloading may be AMPK dephosphorylation. Another possible cause for the decrease in RMP may be a change in the concentration of circulating ouabain [81]. In addition, the decrease in RMP in rat soleus during the first hours of unloading is accompanied by a disruption of lipid rafts in sarcolemma [82,83]. Further investigation is needed to find out whether there are any cause-and-effect relationships between these phenomena. In 2008, we proposed a hypothesis suggesting activation of dihydropyridine receptors (DHPR) in response to the subthreshold depolarization of sarcolemma [76]. The principle possibility of such effect is confirmed by an increase in the influx of calcium ions through DHPR during cell depolarization with micromolar doses of ouabain [84]. The activation of DHPR in addition to the accumulation of calcium ions in the myoplasm can lead to increased permeability of pannexin channels for ATP and activation of purinergic receptors that trigger ROS production by NOX-2 [74]. Thus, ROS produced by NOX-2 (and by neuronal NO-synthase as well, see below) at the early stage of gravitational unloading can initiate rearrangement of several intracellular signaling pathways in the postural soleus muscle fibers and may be considered as metabolic triggers of the atrophic processes. At present, one can certainly state that at later stages of the development of the inactivity-induced muscle atrophy, the production of ROS occurs with significant involvement of the mitochondrial component [70].

In myotube experiments, it was shown that one of the most important factors that stimulate the production of ROS by both mitochondria and NOX-2 is an increase in calcium concentration in myoplasm [28]. The role of disuse-induced calcium accumulation in skeletal muscle fibers has been widely discussed in the literature [25,26]. First of all, calcium ions are discussed as the activators of the calpain-dependent breakdown of cytoskeletal and other functionally significant proteins. By the end of the third day of unloading, the calcium ion accumulation together with a decrease in the levels of NO (see below), a powerful endogenous calpain inhibitor, lead to the activation of µ-calpain [85,86], significant destruction of many important cytoskeletal proteins and subsequent decrease in the intrinsic stiffness of muscle fibers [87,88]. 

In recent years, the role of calcium ions in the regulation of mitochondria and ROS production during unloading is actively discussed [28]. The role of calcium ions as the regulators of gene expression in the initial period of unloading has yet to be determined in future studies. Other important protein kinases activated by calcium ions or calcium/calmodulin complexes include different isoforms of protein kinase C, calcium-calmodulin kinase II and IV, as well as a family of mitogen-activated protein kinases (p38 MAPK, ERK1/2, JNK). An increase in p38 phosphorylation was detected in rat soleus muscle after 3 days of HS [89]. Moreover, this phosphorylation was accompanied by the activation of MuRF-1 expression. Interestingly, an unloading-induced increase in MuRF-1 expression and soleus muscle loss were effectively prevented by the use of a selective p38 inhibitor [89]. It is clear that p38 MAPK activation during the initial period of unloading may be associated with the action of various metabolic triggers, but the role of calcium-dependent mechanisms cannot be excluded.

Thus, during 1–3 days of unloading, we can see energy-dependent dephosphorylation of AMPK, accumulation of reactive oxygen species leading to the IRS-1 degradation and activation of FOXO-dependent expression of U3-ubiquitin ligases, as well as accumulation of resting myoplasmic calcium, which triggers a chain of functionally significant signaling processes. 

## 5. Nitric Oxide: Friend or Foe in Signaling Remodeling? Localization, Traffic, and Activity of nNOS during the Initial Stage of Unloading. Signaling Impact of NO

In recent years, it has become clear that a number of important signaling pathways in skeletal muscle are triggered or modulated by nitric oxide (NO). The role of NO in the regulation of vascular tone and resident stem cell activity in skeletal muscle is well known [90]. It is also known that high concentrations of NO can lead to a decrease in the contractile properties of muscle fibers and calcium sensitivity of myofibrils [91,92]. At the same time, it is well known that NO production significantly increases with physical activity. Suzuki et al., 2007 showed that NO concentration determined by electron paramagnetic resonance (measured in homogenate at room temperature) is significantly increased in murine skeletal muscle following 14-day unloading [93]. At the same time, it is surprising that in this study neuronal NO-synthase (nNOS)-null mice the content of NO in soleus muscle under normal conditions did not differ from that of wild-type mice. The authors of this paper linked the increase in NO production with the translocation of nNOS from the sarcolemmal compartment to the cytosol [93]. Other authors also observed a transition of nNOS molecules from the membrane-bound state to the cytosol during unloading [94,95,96]. Researchers of the Gorza’s group observed this phenomenon after 6 h of HS [21]. However, neither Suzuki et al., 2007 nor other studies have shown a direct relationship between nNOS release to the cytosol and an increase in NO production, which was later noted in several reviews on this issue [19,20]. Moreover, in one study with transgenic dystrophin-deficient mice with nNOS overexpression, it was found that the positive effect of nNOS-dependent gene therapy was not associated with the localization of the newly formed enzyme: cytoplasmic nNOS was as effective as sarcolemmal nNOS [97].

In our laboratory, a significant and deep decrease in NO content in rat soleus muscle was found after 14-day HS [98]. These data were obtained by electron paramagnetic resonance spectrometry (measured in an intact isolated muscle at liquid nitrogen temperature) and were reproducible [99,100]. 

Thus, to date, available data concerning the actual NO amount in the unloaded soleus are not only controversial but also were collected after prolonged periods of disuse (14-day-unloading). It is relevant to note that the technique used there also measured released NO by non-muscle cells, vessels included, and that other approaches, like NO fluorescent traps, are reliable for measurements in intact cells only, therefore not suitable for cryosections [97]. Thus, the actual NO amount after a short unloading remains undetermined. Interestingly, according to the preliminary data, a 24 h unloading can lead to a two-fold decrease in NO content in rat soleus muscle and remain at about the same level at later time-points of unloading (unpublished observations). There are also reports on a decrease in nNOS content during unloading in skeletal muscle of both humans and animals [27,101,102].

It is obvious that the elucidation of this issue requires further experimental research involving other laboratories and methodological approaches. The author of the present review will rely mainly on the data obtained by Lomonosova et al. [98] since these data are in good agreement with evidence on the total nNOS content in soleus muscle during unloading as well as results obtained in experiments with the modulation of NO levels in muscle fibers [99,100,103].

Calpain-dependent proteolysis is considered to be a keyway of nNOS degradation [104]. However, it is clear that during the first hours of unloading, a decrease in NO levels is associated with a decrease in nNOS activity rather than nNOS degradation. To date, several mechanisms of nNOS activation have been described: phosphorylation of one of the IGF1-dependent kinases [105], phosphorylation of AMPK or protein kinase D [106,107], activation of mechanosensory molecules (mechanotransduction) [108,109], as well as activation by an increased concentration of calcium ions [92]. At the same time, the current literature lacks data on the total nNOS content in skeletal muscle at the early stage of gravitational unloading. Therefore, the cause of the unloading-induced NO decrease in a mammalian postural muscle remains unknown.

Before discussing the signaling role of reduced NO levels at the early stages of unloading, it is necessary to consider the data on the atrogenic role of NO reported by a number of authors. For example, Suzuki et al. found that an nNOS-specific inhibitor, 7-nitroindazole, partially prevented HS-induced muscle atrophy in mice [93]. In addition, nNOS-null mice showed milder soleus muscle atrophy than wild-type mice after unloading [93]. At the initial stage of unloading (6–12 h), the blocking of expression/activity of nNOS can lead to a reduced FOXO3 nuclear translocation and subsequent decrease in transcription of genes encoding E3-ubiquitin ligases (MuRF-1 and MAFbx/atrogin-1) [21]. These data are interpreted by the authors as evidence of the early signs of nitrosative stress in the muscle fibers due to increased NO levels. Nitrosative stress (as a type of oxidative stress) is characterized by an accumulation of free radicals that have a direct destructive effect on muscle proteins as well as induce a number of signaling pathways leading to the activation of proteolytic processes in muscle fibers [110]. Indeed, when NO interacts with other ROS, a significant activation of peroxidation processes occurs due to the formation of peroxynitrite, a powerful oxidant that can damage a wide array of molecules [111,112]. However, it should be noted that during longer exposures to unloading, administration of NO donor L-arginine can prevent a number of atrophic and other destructive events in the postural soleus muscle [98,100,103]. It was also shown that the soleus muscle mass recovery after prolonged immobilization is hampered when nNOS activity is inhibited with L-NAME [113].

Data obtained in recent years allow us to make several hypotheses linking the most important signaling events with the changes in NO content observed in skeletal muscle fibers during the initial period of unloading. 

There are several NO-dependent signaling pathways in skeletal muscle fibers. However, as in the case with the energy-dependent signaling, there is, possibly, a NO-sensor molecule that serves as a transmitter of a signal which is determined by the high or low NO levels in muscle fibers. The role of such a molecule can play glycogen synthase kinase 3β (GSK3β). When phosphorylated at Ser9, the enzymatic activity of GSK3β is inhibited.

How do changes in muscle activity affect the regulation of GSK3β activity and, accordingly, the signaling processes in muscle fiber? Several upstream protein kinases mediate GSK3β Ser9 phosphorylation, among which are protein kinases A (PKA) and B (AKT) [114]. However, recently Chibalin et al., 2018 showed an increase in PKA activity in rat soleus muscle after 6 and 12 h of HS [34], so it is unlikely that this protein kinase is responsible for a decrease in inhibitory GSK3β Ser9 phosphorylation at this stage. In addition, a decrease in AKT Ser473 phosphorylation in rat soleus after 24 h of unloading was not prevented by foot mechanostimulation, while a decrease in GSK3β Ser9 phosphorylation was successfully prevented by mechanostimulation [14]. It was found that GSK3β can be phosphorylated on Ser9 by one of the cGMP-dependent protein kinases (most likely by protein kinase G) [115,116]. This cascade is directly dependent on the NO content in the cell. Therefore, it is likely that a decrease in NO content in soleus muscle during the first day of unloading can contribute to the activation of GSK3β and, accordingly, a decrease in the intensity of anabolic processes in muscle fibers. The dependence of GSK3β Ser9 phosphorylation on the NO content under unloading conditions was directly shown by Sharlo et al., 2020 in rat experiment with L-arginine and L-NAME administration during 7-day HS [100]. There is evidence to suggest that there is a connection between GSK3β and NO during the first day of unloading. Moreover, it has recently been shown that GSK3β activity can be suppressed by direct S-S nitrosylation [117].

GSK3β carries out, primarily, inhibiting/blocking signaling functions. Phosphorylation of eukaryotic initiation factor 2B (eIF2B) by GSK3β can lead to a reduction in mRNA translation initiation [118], thereby reducing translation efficiency. GSK3β can also phosphorylate β-catenin, preventing its nuclear translocation and thereby preventing the expression of genes necessary for muscle growth [119]. GSK3β is also able to reduce the intensity of rRNA synthesis through ubiquitylation of proto-oncogene c-Myc, a key regulator of the expression of ribosomal genes [120]. We first found a significant decrease in the rate of protein synthesis and 18S and 28S rRNA content in rat soleus muscle after 24 h of HS, accompanied by a decrease in the content of GSK3β Ser9 phosphorylation indicating an increase in the GSK3β enzymatic activity [14]. GSK3β can also have a negative impact on mitochondrial dynamics. By phosphorylation of transcription factor TFEB, GSK3β can prevent a nuclear translocation of TFEB resulting in inhibiting transcription of the gene encoding PGC1, an important cofactor controlling mitochondrial biogenesis [121]. 

**Figure 5:**
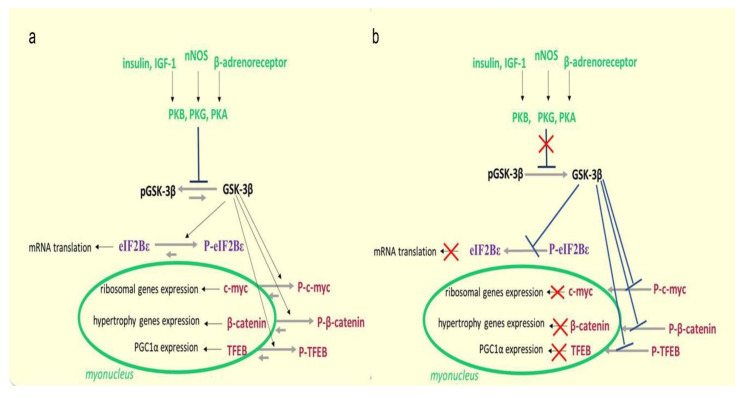
GSK3β as a negative integrator of signaling pathways in muscle fibers and its role under unloading conditions. (**a**) GSK3β in an active skeletal muscle fiber. Following Ser9 phosphorylation by AKT (insulin/IGF-1 - dependent signaling), protein kinase G (NO-synthase/cGMP signaling) or protein kinase A (β-AR/cAMP signaling) GSK3β gets inactivated and does not interfere with 1) initiation of mRNA translation via eIF2Bε, 2) β-catenin, c-Myc and transcription factor TFEB nuclear translocation and, accordingly, expression of hypertrophic genes, 3) the synthesis of ribosomal RNAs and peroxisome proliferator-activated receptor-gamma co-activator 1 (PGC-1). (**b**) GSK3β at the early stage of unloading. Under reduced AKT (Ser473) phosphorylation and NO production, GSK3β (Ser9) phosphorylation is reduced, and GSK3β activity is increased. Therefore, GSK3β inhibits mRNA translation initiation and nuclear translocation of the regulators of transcription and expression of the key genes.

Thus, a decrease in NO content in the soleus muscle at the early stage of unloading can lead to the activation of GSK3β and, accordingly, to a decrease in both translational efficiencies (through eIF2B inactivation) and translational capacity (through c-Myc proteolysis and reduced transcription of ribosomal genes). However, it seems to us that the proposed hypothesis most likely describes the mechanism of a dramatic decrease in the rate of protein synthesis in the soleus muscle during the first day of unloading (Figure 5).

## 6. Mechanosensory Response to the «Activity-to-Disuse» Transition

Direct action of a mechanical stimulus is one of the possible ways of the regulation of NO production in skeletal muscle fibers [108,122]. A number of other signaling pathways in skeletal muscle are also regulated by mechanosensory inputs. 

**Figure 6:**
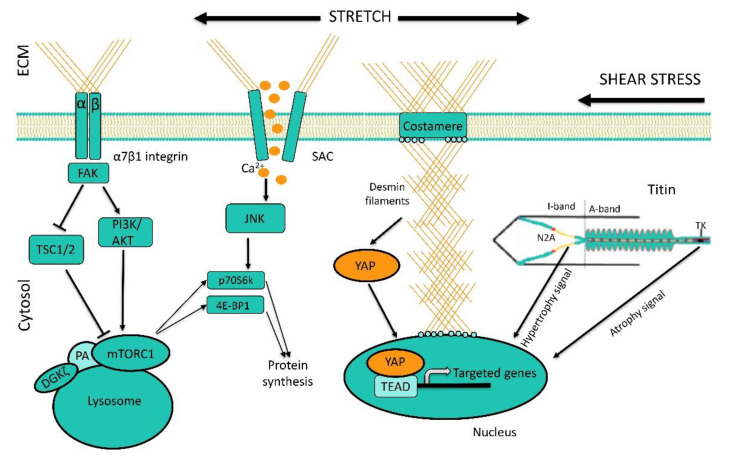
Mechanosensory structures in the muscle fiber. Mechanical factors affect the muscle fiber through a resistive load (the active factor of which is stretching, i.e., an increase in the fiber length relative to its average or minimum length) as well as shear stress, i.e., through the interaction of the fiber with external structures, which move relative to its surface. Integrin molecules, embedded in the sarcolemma, interact with the fibrillar components of the extracellular matrix (ECM) and, when shifted (changing the angle of inclination relative to the membrane), transmit a signal to the focal adhesion kinase (FAK), which, through AKT phosphorylation or TSC2 inhibition, stimulates mTORC1 activity and, consequently, protein synthesis. A change in the fiber can lead to the opening of stretch-activated or mechano-stimulated cation channels allowing for Ca^2+^ influx, and subsequent activation of calcium-dependent kinases transmitting a signal towards ribosomal kinases. An important component of mechanotransduction is the interaction of cytoskeletal molecules (for example, desmin) with Yes-associated protein (YAP), which can translocate to the myonucleus and, interacting with transcription factor TEAD, regulate the expression of a number of key genes. Under mechanical perturbation (shortening or lengthening of the fiber), the giant protein of the sarcomeric cytoskeleton titin releases titin-linked signaling molecules (MuRF-1 and 2, muscle ankyrin repeat protein), which are translocated into the myonucleus and participate in the regulation of gene expression.

A skeletal muscle, as an organ that performs a huge amount of mechanical work, is equipped with a corresponding mechanosensory apparatus that transforms mechanical perturbations into molecular signals involved in the processes regulating muscle plasticity and energetics. Unfortunately, mechanosensory molecules in a skeletal muscle are poorly studied. Mechanosensory molecules can be divided into two main groups: 1) sarcolemmal mechanosensors and 2) sarcomeric mechanosensors. The first group includes stretch-activated ion channels (the molecular basis of which consists of ion channels of the TRPC family and Piezo structures), integrin-associated focal adhesion complexes, and components of the dystrophin-glycoprotein complex (DGC) [123]. Recently, the components of the Hippo signaling pathway have also become a part of mechanosensory elements [124]. The most frequently mentioned mechanical sensor of the sarcomeric apparatus is titin, or rather, several domains of that giant molecule (Figure 6). 

To date, the effects of two types of mechanical perturbations on soleus muscle fibers are most studied. During muscle contraction, muscle fibers slide along the structures of the extracellular matrix resulting in shear stress which is sensed by sarcolemmal mechanosensors. When the soleus muscle has to overcome or hold an external load (bodyweight) during contraction, the sarcomere length exceeds its minimum values creating conditions for a relative stretching of the muscle. This factor can be referred to as the longitudinal loading factor. In vitro myotube experiments have shown that shear stress, but not 15% cyclic strain, can stimulate signaling processes that lead to NO production. It appears that in this case the sensing of mechanical signals is carried out via both integrin-dependent structures (disruption of the glycocalyx prevented shear-stress-induced NO production) and stretch-activated ion channels [108]. It should be noted that in a similar study by Soltow et al. [109], the cyclic stretch of myotubes led to an increase in NO production. In this study, it was shown that there is a stepwise increase in NO production with increasing magnitudes of a stretch from 6 to 18% and only 18% stretch was able to induce a significant increase in NO production in myotubes. This difference in the degree of the applied stretch in these two experiments might explain the apparent discrepancies concerning the NO production.

It is natural to assume that molecular mechanosensors do not experience a routine mechanical stimulation under the lack of muscle contractile activity and, accordingly, mechanical axial loading on the soleus muscle under hindlimb unloading, dry immersion or space flight. It is well known that 7-14-day hindlimb unloading leads to a decrease in the content and kinase activity of focal adhesion kinase (FAK) in rat soleus muscle [125,126]. Unfortunately, there is a lack of data on the changes in the integrin/FAK complex at the early stages of unloading. Given the importance of this signaling hub, future studies will, hopefully, address this important issue. Two-week hindlimb suspension also results in a decrease in the protein levels of TRPC1 and TRPC3 in murine soleus muscle [127]. These proteins are considered to be components of the stretch-activated ion channels [128]. Unfortunately, in the available literature, there is a lack of data on the state of these signaling molecules in skeletal muscles at the early stage of gravitational unloading.

During the study of the effects of unloading on the process of mechanotransduction in skeletal muscle, an interesting phenomenon was revealed: after 24 h of unloading, an anabolic response (i.e., the rate of protein synthesis) of the isolated rat soleus muscle to a bout of eccentric contractions was significantly lower than that of the isolated muscle taken from the control animals [129]. This effect persisted at the later time-points (3 and 7 days) of unloading. This phenomenon could be caused by changes in the functions of mechanosensitive molecules, as well as disturbances in the process of signal transduction. It is known that the activity of stretch-activated (mechanosensitive) channels can be involved in the regulation of anabolic response to mechanical loading. Inhibition of these channels with gadolinium resulted in a significant reduction in p70S6K phosphorylation in response to eccentric contractions [130]. We hypothesized that unloading-induced mechano-anabolic resistance could be associated with the malfunction of the stretch-activated channels. Gadolinium treatment of the isolated rat soleus muscles taken from the intact control animals significantly reduced p70S6K phosphorylation in response to eccentric contractions [129]. At the same time, gadolinium treatment of the isolated muscles taken from the 7-day unloaded rats did not lead to a further decrease in the anabolic response to eccentric contractions [129]. These results indicate that the mechanisms induced by unloading are similar to those elicited by gadolinium. Consequently, the decrease in the amplitude of the anabolic response of the isolated soleus muscle to eccentric contractions after unloading is most likely associated with the impaired function of the stretch-activated ion channels. Although this conclusion is drawn from the 7-day unloading studies, there is no reason to doubt that similar mechanisms (impaired function of the stretch-activated channels) can be implicated in a blunted anabolic response to eccentric contractions at the early stage of unloading.

The nature of the described phenomenon may be related to changes in the microenvironment of mechano-sensitive channels. It has been shown that the function of these channels may be dependent on the content of membrane cholesterol. When cholesterol is removed, the ion flux through mechano-sensitive channels is significantly decreased. However, the ion flux can be restored by the experimental disintegration of the actin stress-fibers network [131,132]. Fluorescence microscopy allowed the authors of these studies to argue that the suppression of stretch-activated channels due to cholesterol removal was associated with the destruction of lipid rafts. Moreover, this effect was directly linked to the reorganization of the network of F-actin stress-fibers, leading to an increase in its rigidity.

At the early stage of gravitational unloading membrane lipid rafts are also disrupted, apparently due to the accumulation of ceramide throughout the sarcolemma [53,54,82]. As a result of the lipid rafts disruption, actin stress-fibers polymerization could create resistance to the mechanical-dependent opening of the stretch-activated channels [133]. Therefore, a decrease in the anabolic signaling response to a mechanical stimulus (mechano-anabolic resistance) may be triggered by the destruction of the membrane lipid rafts.

At the same time, it has been shown that early unloading can lead to the subsarcolemmal cytoskeleton remodeling in rat soleus muscle [134]. The interpretation of these data is slightly different from those outlined above. Ogneva et al., 2014 found a decrease in the content of α-actinin-4 in the membrane fraction of rat soleus muscle after 6 and 12 h of hindlimb suspension. The content of α-actinin-4 in the cytosolic fraction, on the contrary, was increased. At the same time, the content of β- and γ-actin in the membrane fraction was decreased. These changes were accompanied by a decrease in the stiffness of the cortical cytoskeleton measured by atomic force microscopy [134]. Interestingly, the introduction of lecithin into the muscle of unloaded animals prevented these changes [135]. The results of the Ogneva’s group clearly contradict the data related to the consequences of lipid rafts destruction (see above). Future studies, hopefully, will clarify this issue.

Over the years, numerous publications (for example, [136]) have discussed a possible role of titin as a mechanosensor. However, few data directly confirming this assumption have been published. The only evidence of the signaling role of titin was the fact that the denervation of gastrocnemius muscle induced nuclear translocation of E3-ubiquitin ligase MuRF-2, which is known to be associated with titin kinase domain in the M-disk zone [137]. In this case, the serum response factor (SRF), which controls the expression of cytoskeletal proteins, is exported from the nucleus and become ubiquitylated. Later, experiments by Lomonosova et al., 2016 found that another isoform of the same E3-ubiquitin ligase (MuRF-1 isoform) translocates to the nucleus after 3 days of hindlimb suspension [138]. In both cases, it was assumed that titin, with A-domain of which both MuRF isoforms are associated, can change its conformation under unloading conditions, allowing MuRF release and nuclear translocation. Unfortunately, it remains unclear how MuRF isoforms can participate in the regulation of gene expression. The target genes of these regulators remain unknown.

One of the most important properties of titin, which can be associated with its possible mechanosensory function, is the ability to significantly change the stiffness of its spring-like PEVK domain. Since most of the motor units of the soleus muscle are “switched off” when the support and axial loading are withdrawn (see above), it can be assumed that titin’s PEVK domain loses its tension. Recently, in experiments with a denervated murine hemidiaphragm, the signaling properties of muscles taken from mutant mouse models with decreased and increased titin stiffness were compared. Denervation atrophy in this experiment was abated by mechanical muscle stretching resulting in the stimulation of anabolic processes. The anabolic effect of stretching was more pronounced in mice with increased titin stiffness [139]. The authors of this report consider that the anabolic signal, in this case, was transmitted via a special titin-linked muscle ankyrin repeat protein. These researchers also suggest that due to stretching, this protein is released from titin and translocated to the nucleus thereby activating the expression of genes controlling anabolic processes in muscle fibers. So, a mechanical signal induced by muscle stretching could be transformed into a chemical signal that stimulates protein synthesis.

Thus, there are very little data on the changes in the function of mechanosensory molecules at the early stage of gravitational unloading. Unfortunately, in the available literature, we have not been able to find any data concerning contributions of the currently known mechanosensors to the regulation of proteolytic signaling pathways. However, with the cautious interpretation of the first results, we can say that the mechano-anabolic activity of both stretch-activated channels and titin domains is decreased in the inactivated skeletal muscle at the early stage of unloading. This occurs during a rapid decrease in ribosome biogenesis induced by a decline in NO production (and, of course, other factors). 

## 7. How the Decline in Neuromuscular Activity Promotes the Decline in Slow Myosin Expression? The Nuclear Import of Suppressors Is Promoted. The Accumulation of the Stimulators Is Forbidden

One of the most important features of gravitational unloading is a change in the gene expression pattern of the myosin heavy chain (MyHC) slow and fast isoforms. Under weightlessness [11,140,141], bedrest [142], dry immersion [143] or rodent hindlimb unloading [144,145] there is a significant decrease in the percentage of slow muscle fibers (type I) expressing the slow MyHC isoform and an increase in fast fibers expressing the fast MyHC isoforms (IIA, IId/x types and IIB type in rodents) in soleus and lateral head of quadriceps femoris muscles. A recent systematic review by Vikne et al., 2020 provides data from 42 studies involving 451 volunteers who were subject to detraining, unilateral leg suspension (ULLS), and bedrest. The mean type I muscle fiber percentage was significantly reduced after interventions and, conversely, the overall fast fiber percentage increased after reduced muscle activity [146]. This fiber type transformation is considered to be one of the reasons for increased fatigue and, consequently, reduced performance of postural muscles after functional unloading. The transformation is associated with a decrease in the expression of the *myh7* gene, which encodes the slow isoform of MyHC, and an increase in the expression of genes encoding the fast isoforms of MyHC [147]. The main activity-dependent mechanisms controlling the expression of the slow myosin isoform gene are described elsewhere [11,147].

It turned out that gravitational unloading (hindlimb suspension) leads to a very early decrease in the expression of slow myosin mRNA in rat soleus muscle. Reduced expression of both precursor mRNA and mature mRNA of MyHC I(β) was observed after 24 h of hindlimb unloading [15]. These data were confirmed by other authors [16,100,138]. What signaling mechanisms are involved in such a rapid response of the myosin gene to the cessation of the soleus muscle activity?

Of all the mechanisms discussed in connection with the regulation of the *myh7* gene expression, the two mechanisms are currently best studied: (1) the calcineurin/NFAT signaling pathway and (2) the epigenetic mechanism of the promoter inactivation by class IIA histone deacetylases. Both of these mechanisms can be regulated at two levels: at the level of nuclear-cytoplasmic traffic and the level of the affinity to *myh7* gene promoter. Currently, almost all information about the activity-dependent regulation of the above signaling pathways is restricted to the regulation of the nuclear-cytoplasmic traffic of the key mediators.

**Figure 7:**
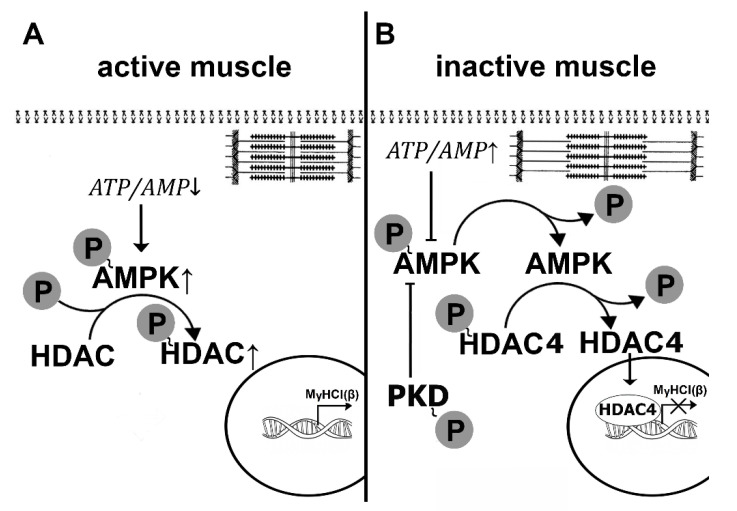
AMPK (Thr172) dephosphorylation as a factor reducing the expression of the slow isoform of MyHC. In active muscle (**A**), phosphorylated (Thr172) and activated AMPK phosphorylates histone deacetylase 4 (HDAC4), preventing its nuclear translocation and thus providing an unhindered expression of the slow MyHC. In the first 24 h of unloading (**B**), changes in the balance of ATP/ADP/AMP and high protein kinase D activity can lead to the dephosphorylation of AMPK, which can no longer prevent HDAC4 nuclear translocation and its interaction with SIRT1, MEF2D, and histone H3. In this case, the expression of the slow isoform of the MyHC gene is inhibited.

The phosphorylation site of histone deacetylase-4 (HDAC4) is combined with the site related to its nuclear transport; hence the phosphorylation of this molecule hampers its myonuclear translocation [148,149,150]. Protein kinases calcium-calmodulin kinase II (CaMKII) and AMPK were shown to phosphorylate HDAC4 [148,149,151,152]. It is known that in fast-type fibers HDAC4 localizes to myonuclei and (together with SIRT-I) deacetylates (and inactivates) transcription factor MEF-2D, which can activate transcription on the promoter of the slow myosin isoform gene [153]. In this case, the transcription of this gene does not occur. It was shown that chronic low-frequency electrical stimulation of the fast-skeletal muscle can lead to an increase in AMP and calcium ions, promoting AMPK and CaMKII activation and resulting in HDAC4 phosphorylation and subsequent expulsion from the myonuclei [152]. In addition, Yoshihara et al., 2016 showed that 10-day hindlimb immobilization resulted in the nuclear accumulation of HDAC4 in rat gastrocnemius muscle which was accompanied by reduced AMPK phosphorylation [154]. As a significant decrease in AMPK phosphorylation was previously detected in rat soleus muscle after 24 h of hindlimb unloading [33], we proposed a hypothesis linking the early decrease in MyHC I(β) expression to AMPK dephosphorylation and subsequent HDAC4 dephosphorylation and nuclear import. To confirm this hypothesis, rats were treated with a selective AMPK activator (AICAR), which allowed us to compensate for a decrease in AMPK (Thr172) phosphorylation in the rat soleus muscle following 24-h unloading [16]. As expected, the content of phosphorylated AMPK and HDAC4 in the nuclear fraction did not differ from control levels and, despite unloading, there was no decrease in the levels of pre-mRNA and mature MyHC I(β) mRNA. The results of the study suggested that a decrease in AMPK phosphorylation that occurs during the first hours of unloading [34] is likely contributing to the dephosphorylation and nuclear import of HDAC4, preventing transcription of the slow myosin gene. Thus, a decrease in AMPK phosphorylation during the first day of unloading due to either a change in the balance of adenine nucleotides or the increase in protein kinase D activity of (see above) can contribute to the decrease in the expression of the slow isoform of MyHC and slow-to-fast transformation of muscle fibers (Figure 7).

Interestingly, 3 days of HS resulted in a decrease in the nuclear HDAC4 content to the control values [155]. It remains unclear which of the protein kinases is responsible for exporting HDAC4 from the nuclear space. It may be 1) AMPK, phosphorylation of which begins to rise [33]), 2) CaMKII, which, probably, becomes more active due to the accumulation of calcium ions in the myoplasm (see above), or 3) protein kinase D, the activity of which is quite high during the initial period of unloading [16]. In addition, the reduction (but not termination) in the expression of the slow MyHC indicates that the blocking of the gene promoter by HDAC4 is not absolute. It is possible that another signaling pathway, calcineurin/NFATc1, participates in this process. This signaling pathway is based on the ability of calcium-dependent phosphatase calcineurin (induced by calcium-calmodulin complex) to dephosphorylate NFATc1 (nuclear factor of activated T cells 1), which is then imported into the nucleus, binds the promoter of the slow myosin gene and activates its transcription (for review see [147]). In muscle fibers, there are at least two proteins that can inhibit calcineurin, calsarcin-2 [156], and MCIP 1.4 (modulatory calcineurin interacting protein) [157]. It is known that MCIP 1.4 expression is regulated by NFATc1, and transcriptional activity of NFATc1 can be assessed by MCIP 1.4 expression [158]. In addition, GSK3β (once more!) can phosphorylate NFATc1 and, thereby, contribute to NFATc1 export from the myonucleus [159].

**Figure 8:**
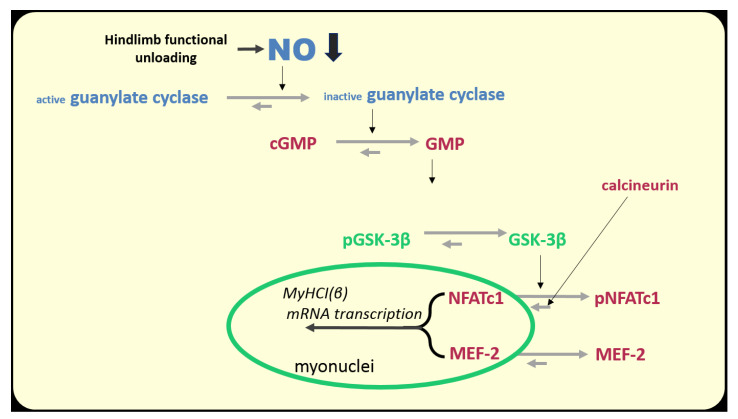
NO-dependent role of GSK-3β in the regulation of nuclear-cytoplasmic traffic of NFATc1 and expression of the slow isoform of the MyHC gene (hypothesis). Rat hindlimb unloading leads to a decrease in NO levels in the soleus muscle, which results in cGMP-dependent GSK-3β dephosphorylation and activation. GSK-3β phosphorylates NFATc1 causing NFATc1 nuclear export, which leads to a decrease in the expression of the slow MyHC gene.

It has been shown that after 24 h of unloading, the content of NFATc1 in myonuclei is decreased by almost 50% compared to the level of vivarium control. It is safe to say that these changes are due to a decrease in the EMG (and, accordingly, contractile) activity of the soleus muscle since daily plantar mechanical stimulation for 4 h maintained NFATc1 nuclear content in myonuclei at the control levels [160]. Interestingly, the transcriptional activity of NFATc1 (determined by MCIP1.4 expression) is decreased by 95% in rat soleus muscle after 24 h of unloading [160]. The cause of the decrease in NFATc1 nuclear content in the rat soleus muscle during the first 24 h of unloading remains unknown. However, it can be assumed that nuclear accumulation of NFATc1 could be limited by its phosphorylation by GSK3β. This assumption is based on the fact that the inhibitory GSK3β Ser9 phosphorylation is reduced by 50% after 24 h of unloading [160]. At the same time, the content of NO in the soleus muscle is also reduced. It is natural to assume that due to the reduced NO production, the activity of cGMP-dependent protein kinase is decreased, which could lead to GSK3β activation and subsequent NFATc1 phosphorylation and export from the myonuclei (Figure 8).

The possibility that NFATc1 can be rapidly exported from myonuclei due to inactivity was supported by the evidence of the low concentrations of NFATc1 and NFATc3 in rat soleus myonuclei during the rest periods of the circadian activity cycle [161]. And, simultaneously, the transcriptional activity of NFATc1, according to the MCIP 1.4 expression, declined much more than NFATc1 nuclear content. In connection with this study, it is worth noting that the state of the NFATc1 nuclear traffic in soleus muscle during the short periods of natural rest is similar to that observed during the early hours of hindlimb unloading. Therefore, the beginning of the disuse period in this regard may be considered as a natural rest period gradually converting to the prolonged inactivity. 

However, it remains completely unclear why a decrease in the transcriptional activity of NFATc1 is much deeper than a decrease in the content of this molecule in the nucleus. It is also unclear how a decrease in the skeletal muscle contractile activity affects the nature of NFATc1 transcriptional activity. In our opinion, the study of the phenomenon of deposition and reversible mobilization of NFATc1 in myonuclei could help, to a certain extent, to solve these issues [162]. It turned out that in skeletal muscle fibers, a significant part of the myonuclear NFATc1 does not bind the promoter of the *myh7* gene, but is stored in the heterochromatin regions, from which NFATc1 can be mobilized to stimulate gene transcription. The process of NFATc1 mobilization may be associated with acetylation and SUMOylation [163,164].

In recent years, a new mechanism regulating the expression of the slow myosin gene via microRNAs has been shown. In mammalian embryonic muscles, as well as in several muscles of other classes of vertebrates, a *myh7b* gene encoding a so-called slow tonic isoform of MyHC is expressed [165]. In the vast majority of mammalian muscles, the expression product of this gene does not exist in the protein form. However, microRNA 499 (miR-499) is encoded by an intron within *myh7b* gene. miR-499 can suppress the expression of a number of constitutive repressors of the MyHCI(β) gene (for example, Sox6) and thereby contributes to the expression of the slow MyHC [166]. In turn, microRNA 208b (miR208b), which is encoded by an intron of *myh7* gene (a slow isoform of MyHC), can stimulate mRNA expression of *myh7b*, which includes miR-499. How this mechanism may be involved in the process of reducing slow myosin expression during the first day of unloading of the postural soleus muscle? 

It has recently been shown that *myh7b* expression in rat soleus muscle after 24 h of unloading is significantly reduced. The reduction in *myh7b* expression was twice as much as that for *myh7* expression [166]. However, plantar mechanical stimulation was able to almost prevent this reduction completely. The decrease in *myh7b* expression was accompanied by a decrease in miR499 (unpublished observation) as well as a bit delayed increase in the expression of the MyHCI(β) repressor Sox6 after 3 days of HS [167]. Thus, we have now encountered a cooperative behavior of the two different genes encoding different slow isoforms of MyHC during support withdrawal-related reduced muscle activity as well as during muscle activity, which is maintained by the mechanical stimulation of the foot. Further research is required to elucidate triggering factors that account for this cooperative activity and determine the expression of which gene is primary.

Thus, a decrease in the expression of the slow isoform of MyHC as one of the central components of the regulatory remodeling of postural muscle molecular structures during the transition from activity to unloading is carried out by several signaling mechanisms, apparently interacting with each other (although the nature of the interaction is still unclear). These signaling mechanisms are triggered by the same messengers (adenine nucleotides, NO, calcium ions) as for the system of protein turnover following the cessation of muscle contractile activity. At the same time, these regulatory mechanisms usually work within two main regulatory circuits: 1) the regulation of nuclear-cytoplasmic traffic and 2) the regulation of affinity to the gene promoter and transcriptional activity itself. Mechanisms underlying the second circuit of regulation are currently vaguely defined.

## 8. Conclusions. How Rest Transforms into Disuse, and how Muscle Senses this Transformation

In summary, when ground reaction force and axial loading are eliminated or reduced, the constant electrical and contractile activity of the postural soleus muscle sharply decreases or ceases. Thus, most of the muscle motor units convert from a state of activity to a state of rest. However, under real or simulated weightlessness the duration of the rest state exceeds the duration of the typical rest state seen under natural conditions. We can assume that in this case, the state of rest gradually converts to the state of disuse. As follows from yet few studies, during this transition period, a rapid rearrangement of various signaling pathways occurs, which, primarily, results in the downregulation of those pathways that play an important role in the maintenance of constant muscle activity. These molecular events lead to a decrease in the rate of protein synthesis (primarily via inhibition of ribosomal biogenesis and activation of endogenous inhibitors of mRNA translation, such as GSK3β) and an increase in proteolysis (for example, via upregulation of muscle-specific E3-ubiqutin ligases). In addition, the expression of a slow isoform of MyHC decreases, and the expression of fast isoforms of MyHC increases. Currently, available data on possible signaling molecules that could trigger this rearrangement of signaling pathways is limited. There is some evidence to suggest that alternations in the concentrations of substrates and products of the routine muscle activity may serve as signals that trigger signaling rearrangements when muscle activity is ceased (for example, an accumulation of ATP and glycogen and a decrease in AMP concentration). Some experimental data supporting this hypothesis were provided in the present review. Intensive NO production in skeletal muscle is usually observed with increased contractile activity [90]. Therefore, it would be natural to expect a decrease in NO concentration when skeletal muscle stops working. In this case, NO can be considered as a potential trigger molecule. Experimental data supporting a hypothesis about a triggering role of reduced NO content in skeletal muscle at the early stage of mechanical unloading were described in the current review. Moreover, there are more experimental data related to the role of NO during longer periods of unloading (see review [20]). In addition, there is evidence that an accumulation of ceramide in the sarcolemma [54] and a decrease in the electrogenic function of Na^+^, K^+^-ATPase [79] may also play a triggering role in these signaling events. As for the accumulation of ROS, their triggering role in the activation of the expression of E3-ubiquitin ligases can be considered proven [67]. Although the source of ROS at the early stage of unloading remains unknown, it is clear that the duration of the maintaining high ROS concentration during early unloading is significantly longer than under various modes of increased muscle contractile activity. With some caution, it can be suggested that, during the first day of unloading, changes in the concentration of adenine nucleotides, glycogen accumulation and an increase in ROS concentration (including NO-associated oxidants) trigger signaling cascades leading to the upregulation of E3-ubiquitin ligases (for example, MuRF1), while a decrease in NO content is generally associated with the downregulation of anabolic processes. All the above-mentioned triggering mechanisms (possibly, in concert with other unknown pathways) are involved in the processes of myosin phenotype transition in the soleus muscle at the earliest stage of unloading.

It is known that by the end of the third day of unloading, the intracellular concentration of calcium ions in soleus muscle fiber increases [24]. An increase in the concentration of calcium ions affects many signaling pathways during this period. However, the phenomenon of calcium accumulation is insufficiently studied, and elucidation of these intriguing mechanisms is the subject of future research.

The current state of the art already shows that the rearrangement of signaling pathways in the postural muscle fibers due to the cessation of muscle activity as well as functions of the molecules triggering this rearrangement unfold gradually over time with certain temporal changes. Monitoring these time-course alternations is essential for researchers in the field.

## Abbreviations

EMG	electromyography
AMPK	AMP-activated protein kinase
GSK-3β	glycogen synthase kinase-3β
HS	hindlimb suspension
MuRF-1	muscle RING finger—1 E3 ubiquitin ligase
MAFbx/atrogin-1	muscle atrophy F-box/atrogin-1 E3 ubiquitin ligase
MyHC I (β)	myosin heavy chain isoform I (β)
ROS	reactive oxygen species
nNOS	neuronal nitric oxide synthase
SAC	stretch-activated ion channel
β-GPA	beta-guanidinopropionic acid
PKD	protein kinase D
p70S6K	ribosomal protein S6 kinase p70
mTORC1	mammalian/mechanistic target of rapamycin
eEF2	eukaryotic elongation factor-2
AICAR	5-amino-imidazole-4-carboxamide ribonucleotide (AMPK activator)
IRS-1	insulin receptor substrate-1
FOXO3A	forkhead box O3A—signaling protein and transcription factor
HDAC5	histone deacetylase 5
cbl-b	Casitas B-lineage lymphoma-b ubiquitin ligase
AKT	protein kinase A
NOX-2	NADPH-oxidase-2
RMP	resting membrane potential
DHPR	dihydropyridine receptor
p38 MAPK	p38 mitogen-activated protein kinase
ERK1/2	extracellular signal response kinase 1/2
JNK	c-jun N-terminal kinase
NO	nitric oxide
L-NAME	N(gamma)-nitro-L-arginine methyl ester—NOS inhibitor
eIF2B	eukaryotic initiation factor 2B
TFEB	transcription factor EB
PKA	protein kinase A
cGMP	cyclic guanosine monophosphate
c-Myc	proto-oncogene c-Myc
DGC	dystrophin glycoprotein complex
FAK	focal adhesion kinase
TRPC1	transient receptor potential channel 1
TRPC3	transient receptor potential channel 3
ULLS	unilateral lower limb suspension
NFAT	nuclear factor of activated T-cells
HDAC4	histone deacetylase 4
CaMKII	calcium-calmodulin kinase II
MCIP 1.4	modulatory calcineurin-interacting protein 1.4
SUMO	small ubiquitin-like modifier (protein)
miR-499	microRNA-499
Sox6	promotor suppressor Sox6
miR208b	microRNA-208b
